# Enhanced Simulation of Infrared Heating of Thermoplastic Composites Prior to Forming under Consideration of Anisotropic Thermal Conductivity and Deconsolidation by Means of Novel Physical Material Models

**DOI:** 10.3390/polym14163331

**Published:** 2022-08-16

**Authors:** Manuel Längauer, Gernot Zitzenbacher, Hannes Stadler, Christoph Hochenauer

**Affiliations:** 1 School of Engineering, University of Applied Sciences Upper Austria, Stelzhamerstraße 23, 4600 Wels, Austria; 2 Transfercenter fuer Kunststofftechnik GmbH, Franz-Fritsch-Straße 11, 4600 Wels, Austria; 3 Institute of Thermal Engineering, Graz University of Technology, Inffeldgasse 25 b, 8010 Graz, Austria

**Keywords:** process modeling, thermoplastic composites, deconsolidation, anisotropic thermal conductivity, CFD

## Abstract

In recent years, thermoplastic composites have found their place in large business sectors and are in direct rivalry to thermoset matrix composites. In order to ensure efficient and lean processes, process modeling gains ever-growing attention. This work shows the computational fluid dynamics (CFD)-modeling of a typical heating step in a thermoforming process of a thermoplastic composite sheet. When heating thermoplastic composites, the heat conduction proceeds anisotropic, and the sheets are subject to thermal deconsolidation when heated above the melting temperature of the polymer matrix adding to the anisotropic effect. These effects are neglected in known process models and this study shows the first successful attempt at introducing them into CFD-modeling of the heating of thermoplastic composite sheets. Thus, the simulation requires temperature dependent values for the anisotropic thermal conductivity and the coefficient of linear thermal expansion, which are calculated with novel physical models which were developed solely for this cause. This alters the behavior of an isotropic CFD-model and allows the successful validation via laboratory experiments using glass fiber reinforced polypropylene (PP/GF) sheets with embedded thermocouples to check the internal temperature distribution when the sheet is heated to the designated forming temperature in a composite thermoforming press. The incorporation of this newly developed process model reduces the error in the core temperature prediction from close to 70 °C to 3 °C at the forming temperature.

## 1. Introduction

Manufacturing of parts from thermoplastic matrix composites and the process modeling thereof has been in the scientific spotlight in the last couple of years. In particular, their high strength, simple processability, and high wear resistance make them very attractive [1,2,3]. The lightweight industry has a huge demand for recyclable, high performance materials to replace metal and thermoset materials. To meet those demands, high standards are imposed on material quality and lean, efficient processes. Many parts are manufactured in stamp-forming processes which are a kind of thermoforming process, which in turn is adopted from thermoplastic processing [4,5,6,7,8]. Therein semifinished sheets are transported to a heating station—mostly infrared heating—to be heated above the transition temperature of the polymeric matrix to make the material formable [9]. This is accompanied by thermal deconsolidation and the volume of the sheet increases significantly, which again coincides with a decrease in the thermal conductivity [10,11,12,13,14,15,16]. When the desired temperature is reached, the sheets are transported to a two-sided mold and formed into a three-dimensional part, cooled down, and demolded.

While much effort has been made on this matter already, especially the heating step in thermoforming processes is still a topic that requires significant work in both academic and industrial prospects. This step is vital, both for the process efficiency since it is the limiting factor when considering cycle time, and also for the quality of the finished product as the temperature and its distribution predetermines the forming behavior of the sheet.

Models for the anisotropic thermal conductivity are available and can be used for heating simulations. Ever wider spreading access to CFD methods has simplified the modeling of the heating behavior and has led to a rise in attempts thereof.

Brogan and Monaghan conducted extensive research on the heating behavior of carbon fiber reinforced polyether ether ketone (PEEK/CF) and proposed an attempt to calculate the temperature distribution when using quartz heaters [17].

One of the first visualized finite element simulations was performed by Johnson and Pickett [18] who investigated double curvature forming of carbon fiber reinforced polyetherimide (PEI/CF). In the same year, Sweeney et al. [19] conducted a study on the heating of PEEK/CF considering different heater setups and composite layups. They concluded that heater-to-composite distance had the most significant effect on the resulting temperature distribution.

Hwang and Hwang [20] used a right angle die to study the forming behavior of carbon fiber reinforced polyamide 6 (PA 6/CF) parts at different heating setups of the laminate. They studied the defects of overheated and underheated parts with micrographs and load tests and concluded that the forming temperature had a larger effect than holding time or heater distance.

Hsiao and Kikuchi (PEEK/CF), Abbassi et al. (carbon fiber reinforced polyphenylene sulfide (PPS/CF)), Chen et al. (PPS/CF) as well as Harrison, Gomes, and Curado-Correia (PP/GF) published numerical studies for the forming of already heated sheets [21,22,23,24]. This approach was also chosen by Stamopoulos and Di Ilio [25] and D’Emilia et al. [26] who both studied the forming of semi-spherical preheated composite parts with the use of numerical and analytical methods.

McCool et al. also worked with PPS/CF and described thermal deconsolidation in the heating stage when reporting the effect of forming and mold temperature on the properties of the formed sheets [27].

Nardi and Sinke [28] calculated the center temperature of a composite sheet analytically with the knowledge of the heater and the initial sheet temperature, the specific heat capacity, the density, and an isotropic thermal conductivity parameter. They considered the whole process and made estimations of the mechanical behavior of the final parts.

This work is a follow-up to preceding studies, in which a model for the anisotropic thermal conductivity of neat consolidated composite sheets was formulated [29]. This model was then refined for thermal deconsolidation, which occurs at temperatures greater than the main transition temperature of the polymeric matrix (melting temperature for PP) [30].

As shown above, most authors presume isotropic properties or isothermal conditions, which is not the case in industrial processes. This study highlights the importance of including temperature dependent anisotropic thermal conductivity and deconsolidation when simulating the heating step in thermoforming of thermoplastic composites with the help of CFD methods. For this reason, heating experiments in a laboratory thermoforming press are performed with varying heater power, heater-sheet distance, sheet thicknesses, and heater setups. Moreover, a CFD model of the process is created, and three different sets of material properties are used as input data. Starting with a very simple isothermal and isotropic model and increasing the complexity to the point at which the anisotropic thermal conductivity, the anisotropic coefficient of linear thermal expansion and the specific heat capacity at constant pressure are temperature dependent. Including these data in a CFD-model for the heating of thermoplastic composites is a novelty to the field. It is shown that the temperature distribution in the sheets can only be predicted at a satisfactory level when the highest level of complexity is used. Simple, isotropic models are proven to be inaccurate and unusable.

## 2. Materials and Methods

In order to examine the infrared heating behavior of thermoplastic composite sheets, it was necessary to manufacture sheets with embedded thermocouples. The chosen material were pre-consolidated PP/GF sheets type Tepex dynalite 104 RG600(4)/47% with a thickness of 2 mm from LANXESS Deutschland GmbH, Cologne, Germany. Those sheets are made up of 2/2 twill-woven glass fibers, embedded in a polypropylene matrix system.

**Figure 1:**
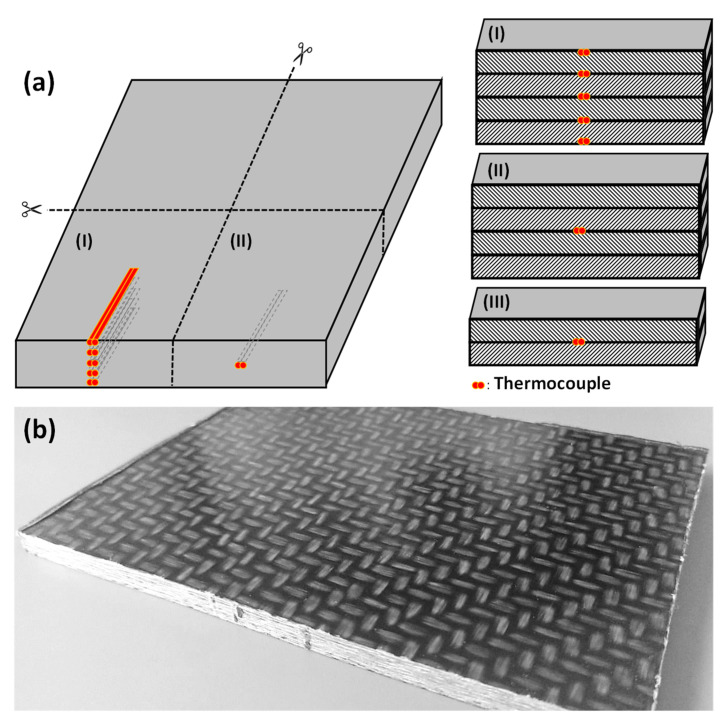
(**a**) Manufactured sheet (**left**) and different sample cross-sections with embedded thermocouples (**right**). The areas are cross-hatched to enable telling apart different layers of stacked sheets. (I) a sample with 5 thermocouples, (II) a sample with one central thermocouple, and (III) a sample made of two sheets with one central thermocouple. (**b**) depicts a sheet of type (II).

Those sheets were stacked in a parallel plate mold (350 mm × 250 mm) with varying numbers of thermocouples in between the layers and/or on the top and bottom surfaces. Since the sheets were later cut to four smaller samples, the thermocouples were placed correspondingly, as shown in Figure 1. The samples (II) in the figure are used to double-check the results from heating experiments with samples (I) and therefore only exhibit one embedded thermocouple. Furthermore, several samples with only 2 stacked sheets (Figure 1a(III)) were manufactured.

The manufacturing of sheets containing thermocouples at a known position was essential for this study and yet, this was the most difficult task since the mold is almost perfectly sealed when closed which led to many broken thermocouple-cables and unusable composites sheets. The sealing is necessary to keep the matrix from being pressed out of the mold and to keep the fiber volume fraction constant.

The pressing of the thick sheets, inspired by the works of Kiss et al. [31,32,33] was performed in a two-stage process using a heating press Wickert WLP 80/4/3 (Wickert Maschinenbau GmbH, Landau in der Pfalz, Germany) and a cooling press Höfer H10 (Höfer Presstechnik GmbH, Taiskirchen, Austria) which are connected by a mold transfer shuttle system.

Temperatures were monitored during every impregnation trial to guarantee equal processing conditions with the thin-film thermocouples type-K, 402–716 (TC Mess-und Regeltechnik GmbH, Mönchengladbach, Germany) and the heating and cooling processes were logged with a Picotech TC-08 logger (Pico Technology, St. Neots, United Kingdom). The following steps were necessary to fabricate the samples:

- Heating the hot press to 300 °C;
- Insertion of the mold containing the stack and applying a pressure of 2 bar;
- Once the thermocouples read a temperature of over 165 °C, the pressure was increased to 10 bar and held until a temperature of 190 °C is reached;
- The hot press was opened and the mold was transferred to the cooling press;
- The mold was cooled to a temperature below 100 °C at a pressure of 5 bar;
- The sheets were demolded using ejector pins.

The sheets were then cut to receive the final parts with 175 mm × 120 mm.

**Figure 2:**
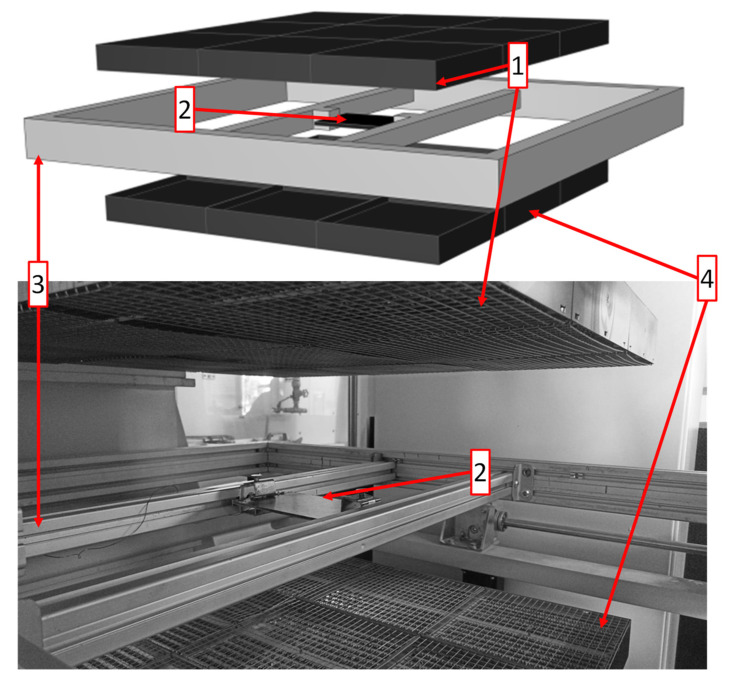
Model of the heating station of the thermoforming device (**top**) and a photograph of the real heating station (**bottom**), where (1) is the upper heater bank, (2) is the thermoplastic composite sample, (3) is the clamping frame, and (4) is the lower heater bank.

The heating experiments were conducted using a thermoforming press type LZT-OK-220-L (Langzauner GmbH, Lambrechten, Austria). This press was equipped with double-sided infrared heating utilizing a total of 18 IR-heaters type Krelus MINI G14-25 M (Leister Technologies AG, Sarnen, Switzerland) with a maximum power of 2.5 kW each. The heaters were vertically adjustable in a range of 140 mm to 440 mm from each other and the maximum power can be adjusted individually. Moreover, the press utilizes a shuttle system in which the semifinished parts were clamped in order to transfer the sheets from the press to the heating station and back to the press when the forming temperature was reached. This work deals solely with the heating step and forming was not considered. The setup is depicted in Figure 2.

The sheets were first clamped into the shuttle frame and the thermocouples were connected to the logger and a semiautomatic process was started:

(1) Transfer the sheet to the heating station;
(2) Activate infrared heating at a predefined power level for a set amount of time;
(3) Shut off heaters and transfer the sheet to the molding station;
(4) Activate convectional cooling.

The temperature of the infrared heaters was determined using an infrared camera system type Optris Xi 400 (Optrix GmbH, Holzkirchen, Germany). One single heater was activated at different power settings and the mean surface temperature was recorded until a steady state was reached.

## 3. CFD Modeling

The Model was created in CATIA V5 2012 (Dassault Systèmes, Vélizy-Villacoublay, France) and mimics the heating station of the thermoforming machine. Figure 2 shows the used model featuring 18 individual heaters of 250 by 250 mm, the clamping frame and the thermoplastic composite sheet.

This model is then exported to ANSYS Workbench 2021 R2 (Ansys Inc., Canonsburg, PA, USA). This tool supports a wide range of customized materials and heating mechanisms. For this work, three different thermal material behaviors were studied. They differ in their linear thermal expansion, specific heat capacity and thermal conductivity. For further reference, the naming and general physical behavior are summarized in Table 1.

The calculation of the anisotropic thermal conductivity was presented in detail in preceding studies of Längauer et al. [29,30]. The basic principle is to define a unit cell of the composite sheet with an edge length *a* and a roving width *b* from which the thermal material properties are calculated. The fibers are considered to be located in a “fiber-rich” layer with the thickness *s*_1_, that is unharmed by thermal deconsolidation. The “matrix-rich” layer with the thickness *s*_2_ is subject to deconsolidation and allows parallelly and serially conducting voids to form above the transition temperature. The parallelly conducting voids are located within the “matrix-rich” layer and therefore increase its thickness with rising temperature. The serially conducting voids form a layer of their own with the thickness *s_v_*. The total void volume is calculated from the temperature dependent fiber volume fraction φ(T) that is derived numerically. Basically, the total volume of the unit cell $V_{T}\left(T\right)$ is increasing with rising temperature

(1) $$V_{T}\left(T\right)=a\left(1+\alpha_{x}\Delta T\right)a\left(1+\alpha_{y}\Delta T\right)s_{T}\left(1+\alpha_{z}\Delta T\right),$$

where $\alpha_{x,y,z}$ are the coefficients of linear thermal expansion in the directions of space, $V_{T}\left(T\right)$ is the temperature dependent total volume of the unit cell, $s_{T}$ is the total unit cell thickness and ΔT is the temperature difference. The thermal expansion in both fiber directions was assumed to be constant and limited to that of glass fibers [31]. The coefficient of linear thermal expansion in transversal direction $\alpha_{z}$ for the *anisotropic and deconsolidating* case is therefore calculated as

(2) $$\alpha_{z}=\frac{1}{\Delta T}\left(\frac{V_{T}\left(T\right)}{{\left(a\left(1+\alpha_{F}\Delta T\right)\right)}^{2}s_{T}}-1\right)\;,$$

With

(3) $$V_{T}\left(T\right)=\frac{V_{F}\left(T\right)}{\varphi \left(T\right)}$$

Here, $V_{F}\left(T\right)$ is the temperature dependent fiber volume which is determined as

(4) $$V_{F}\left(T\right)=\frac{m_{A}a^{2}}{\rho_{F}}{\left(1+\alpha_{F}\Delta T\right)}^{3},$$

where $m_{A}$ is the area density of the textile and $\rho_{F}$ is the fiber density.

**Figure 3:**
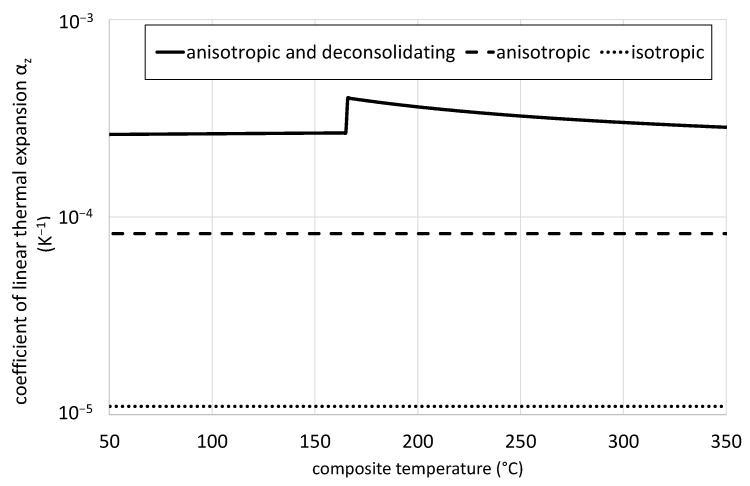
Transversal coefficient of linear thermal expansion $\alpha_{z}$ for *anisotropic and deconsolidating* composites and *isotropic* materials with isothermal behavior.

The exact input data for the transversal coefficient of linear thermal expansion is sketched in Figure 3. The *isotropic* case considers an isothermal coefficient in all directions of space. This value is provided by the material datasheet. The transversal coefficient of linear expansion for the *anisotropic* material was set to 8.25 × 10^−5^ K^−1^, which corresponds to the mean value of the coefficients of a typical PP grade and glass fibers [34,35]. The transversal coefficient of linear expansion of the *anisotropic and deconsolidating* material according to Equation (2) exhibits a rapid increase at the melting temperature of the polymeric matrix and an almost steady decline from there. This of course has an impact on the density function that is required for the thermal simulation.

Following [29], the thermal conductivity in the fiber direction $k_{∥}$ for the *anisotropic* case was calculated as

(5) $$k_{∥}=\frac{\left(A_{F,2∥}k_{F}+A_{P,2∥}k_{P}\right)}{1+\frac{a-b}{b}\cdot \frac{\left(A_{F,2∥}k_{F}+A_{P,2∥}k_{P}\right)}{\left(A_{F,1∥}k_{F}+A_{P,1∥}k_{P}\right)}}\frac{1}{\left(s_{1}+s_{2}\right)\cdot b},$$

where *k_P_* and *k_F_* are the thermal conductivity of the polymer and the fiber material, respectively and *A_F_* and *A_P_* are the corresponding areas of fiber and polymer, respectively, in each layer (with thickness *s*_1_ and *s*_2_), as the indices 1 and 2 imply [29].

In the preceding study [30], it was concluded that the fiber material is the dominant factor for heat conduction and that thermal deconsolidation cannot break the fiber network. For this reason, the thermal conductivity in the fiber direction is constant over the deconsolidation temperature resulting in the same values for $k_{∥}$ for the anisotropic and the *anisotropic and deconsolidating* case.

According to [30] for the transversal direction, the thermal conductivity $k_{⊥}$ for the *anisotropic* case is calculated as

(6) $$k_{⊥}=\frac{s_{1}+s_{2}}{\;s_{2}}\cdot \frac{k_{P}}{1+\frac{s_{1}}{s_{2}}\cdot \frac{k_{P}a^{2}}{A_{P1⊥}k_{P}+A_{F⊥}k_{F}}},$$

where *A_P_* and *A_F_* are again the corresponding areas of polymer and fiber, respectively, and the index 1 stands for the “fiber-rich” layer. When thermal deconsolidation takes place, a new void layer is formed, and the “matrix-rich” layer expands leading to a temperature dependent transversal thermal conductivity $k_{⊥}\left(T\right)$

(7) $$k_{⊥}\left(T\right)=\frac{k_{v}\;k_{parallel}\left(s_{1}+s_{2}+\frac{V_{v}\;T}{T_{0}a^{2}}+s_{v}\right)}{k_{v}\left(s_{1}+s_{2}+\frac{V_{v}\;T}{T_{0}a^{2}}\right)+k_{parallel}s_{v}},$$

where *V_v_* is the initial void volume, *T*_0_ is the initial temperature, *k_v_* is the thermal conductivity of the voids and $k_{parallel}$ is the thermal conductivity in the original unit cell, considering the corresponding areas of voids, fibers and matrix but neglecting the newly created void layer.

(8) $$k_{parallel}=\frac{(A_{P2}k_{P}+A_{v}k_{v})\left(A_{F}k_{F}+A_{P1}k_{P}\right)\left(s_{1}+s_{2}+\frac{V_{v}\;T}{T_{0}a^{2}}\right)}{(A_{P2}k_{P}+A_{v}k_{v})a^{2}s_{1}+a^{2}(A_{F}k_{F}+A_{P1}k_{P})\left(s_{2}+\frac{V_{v}\;T}{T_{0}a^{2}}\right)}.$$

The thickness of the void layer $s_{v}$ is derived from the subtraction of the void volume increase in layer 2 given by the general gas equation from the total expansion calculated from the temperature dependent fiber volume fraction and the initial fiber volume fraction $\varphi_{0}$

(9) $$s_{v}=\frac{m_{A}}{\rho_{f}}\left(\frac{1}{\varphi \left(T\right)}-\frac{1}{\varphi_{0}}\right)-\frac{V_{v}\;T}{T_{0}a^{2}}$$

**Figure 4:**
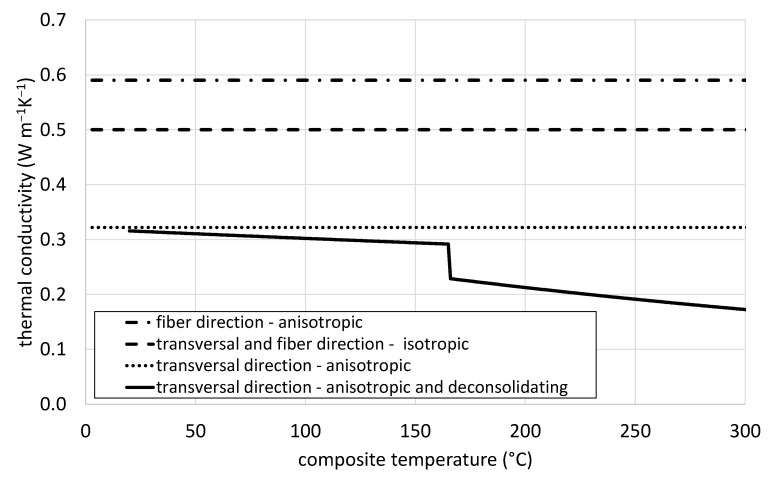
Temperature dependent thermal conductivity used in the CFD models for each specific material behavior.

Figure 4 shows the input data for the thermal conductivity. As one can see, *PP/GF isotropic* uses constant values for all directions. The *anisotropic* case is a bit more advanced and uses values from Equations (5) and (6) for the anisotropic thermal conductivity, distinguishing between the fiber and the transversal direction. The input data which is also used for *anisotropic and deconsolidating* is provided in Table 2. Equation (7) is utilized to receive the transversal thermal conductivity for the *anisotropic and deconsolidating* case in which there is a steady decline until reaching the melting point of the matrix material where a drop in the thermal conductivity is visible due to the rapid void growth and creation. From there the transversal thermal conductivity is declining in a linear fashion again. 

The temperature dependent specific heat capacity was previously tested and is derived from [29]. The isothermal value for the specific heat capacity at constant pressure of the *isotropic* material was set to 1.01 J·g^−1^·K^−1^, since this is the rounded measuring value at 30 °C.

**Figure 5:**
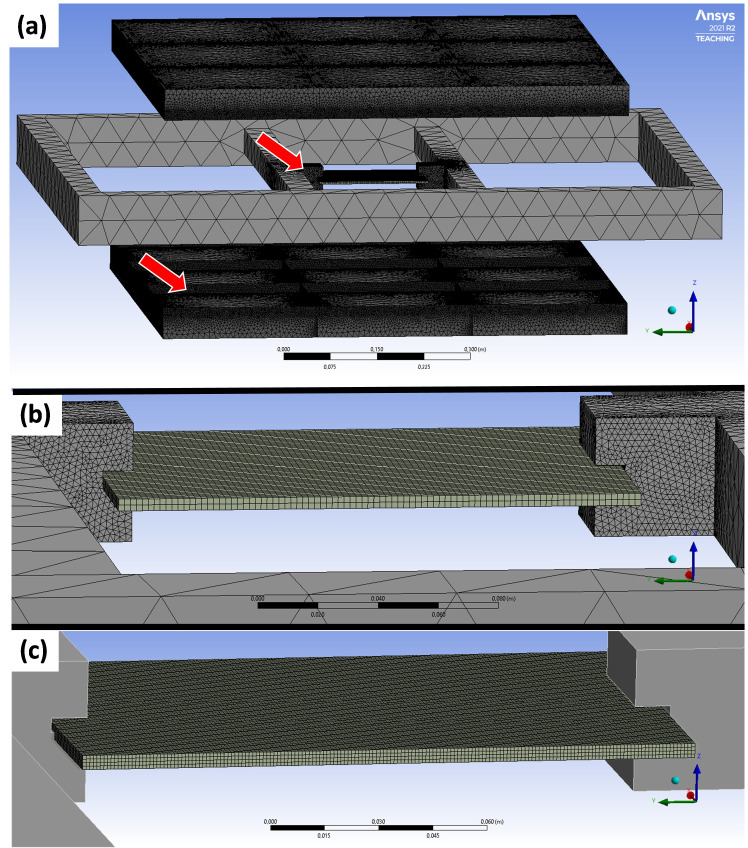
(**a**) Meshed model in ANSYS Workbench 2021 R2 (the red arrows indicate areas with finer face meshing) with a detailed view of the meshed, clamped thermoplastic composite sheet in (**b**) and a finer meshed model for the sheet with a suppressed mesh at the clamping frame (**c**).

The resulting values were provided to the *engineering data* for each material. In a following step, the transient heating evaluation was performed using surface to surface radiation from the heaters to the sheet (emissivity of one). Furthermore, convection in the heating station was considered at all sheet surfaces. The heat transfer coefficient was calculated according to [9] and is between 3.6 W·m^−2^·K^−1^ and 10.6 W·m^−2^·K^−1^ in the relevant temperature range of 30 °C to 300 °C. The meshing of the heaters and the aluminum clamping frame was uncritical, so a maximum element size of 5 cm and auto fitting was used (Figure 5a). In order to minimize the skewness in critical areas, *face* *sizing* with an element size of 2 mm was used. This is clearly visible at the clamps of the frame and the reflectors of the heaters (red arrows in Figure 5a). The sheet itself was meshed with *body sizing*, cartesian elements and quadratic element order with a maximum edge length of 2 mm to provide sufficient data (Figure 5b). Moreover, a comparison with an element size of 1 mm was made (Figure 5c).

Table 3 contains the information about the different components of the model and their mesh statistics. The skewness is supposed to be smaller than 0.9, the element quality as well as the aspect ratio should be close to 1. At the heaters and the clamping frame, which are both not as relevant for the simulation as the sheet, the average values for those parameters are in an acceptable range. The sheet was meshed twice with differing element count. Yet, Table 3 shows that the mesh statistics are almost the same for both cases with only minor deviations. 

The heating was simulated for at least 90 s, and as a result the total temperature distribution of the part and the temperature of the measuring spots on and in the sheet was received for each condition. This was accomplished by placing temperature probes in the model at local coordinate systems.

**Figure 6:**
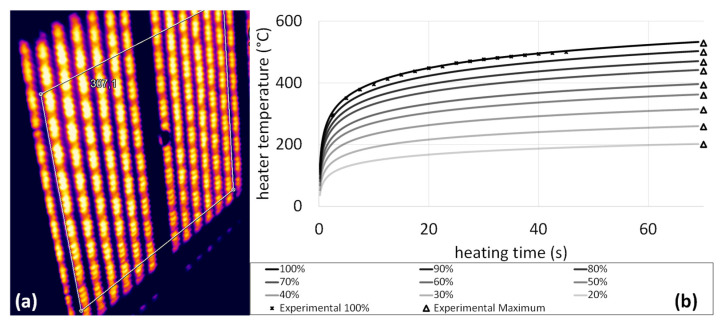
(**a**) Infrared camera image of a single heater. The white frame shows the region of interest, that was selected to determine the mean heater temperature. (**b**) Fitted heater temperature over the heating time with varying heater settings including one experimental series over time and the maximum measured mean heater temperature for each condition.

The temperature of the heating elements was calculated according to Equation (4) for each heater setting. Figure 6 shows the results of the temperature readings of the heating tests of the single heater in the laboratory thermoforming press at different conditions over time. The infrared camera records one reading for each pixel. Yet, in the simulation a mean value for the heater temperature is used for the infrared radiation heating which is mimicked by averaging over a large area on the heater surface—even the unheated crosspiece in the center. 

A logarithmic fit with the parameters *C* and *D* was applied to calculate the heater temperature *T_H_* over the time *t*

(10) $$T_{H}=C\ln\left(t\right)+D$$

Table 4 shows the used fit parameters for various heater settings. 

## 4. Results

**Figure 7:**
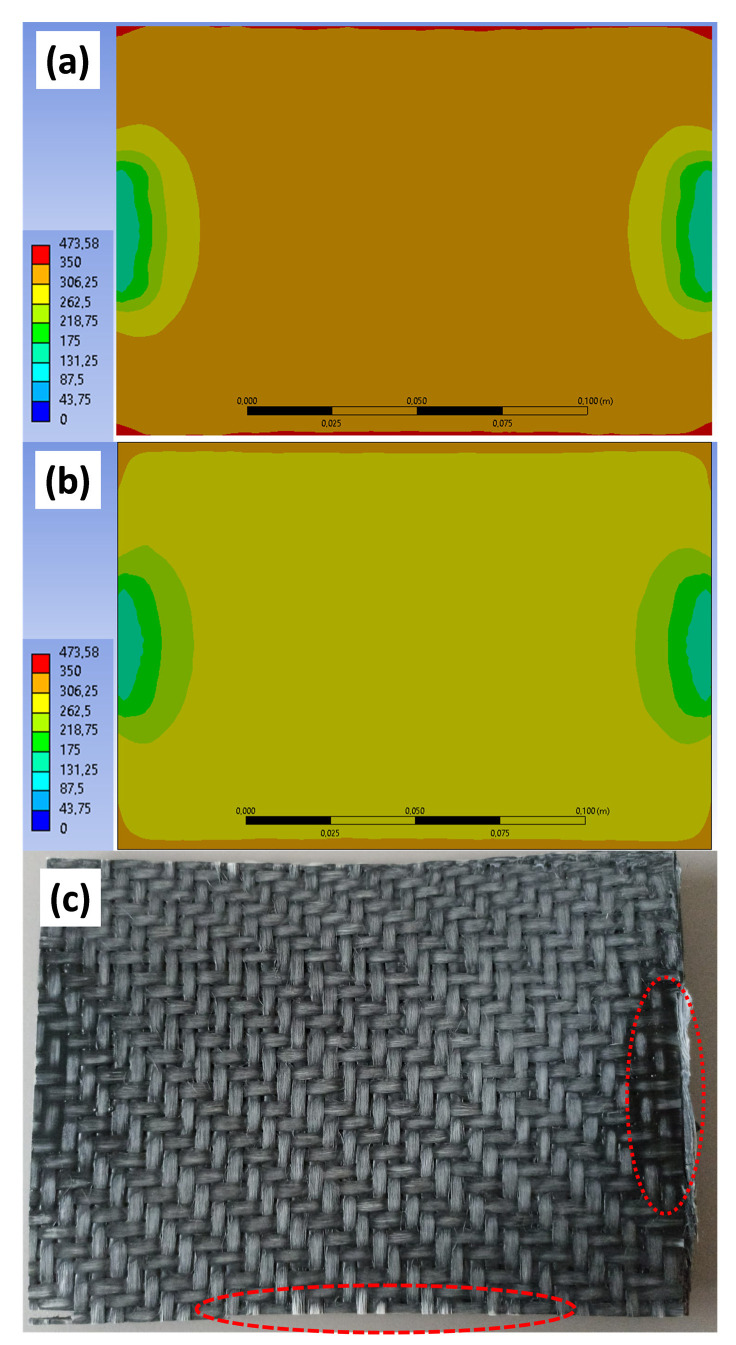
Surface temperature distribution after 100 s of heating with a power of 80% at a distance of 150 mm in case of an *isotropic* sheet of 4 mm thickness (**a**) and an *anisotropic and deconsolidating* sheet of 4 mm thickness (**b**) in comparison to a real composite sheet that has been subjected to these heating conditions (**c**). The dashed area indicates signs of overheating whereas the dotted area is still consolidated.

The heating tests at the laboratory thermoforming station revealed a very inhomogeneous temperature distribution over the sheet thickness but also at the sheet surfaces. This can also be seen in Figure 7, where a comparison of the simulated sheet surface between *isotropic* (a) and *anisotropic and deconsolidating* (b) material behavior is made. The *isotropic* case reacts in a more homogenous way to the temperature increase but appears overheated in almost all areas (T > 300 °C). Only in the areas in and surrounding the clamps the temperature is lower. The *anisotropic and deconsolidating* case shows signs of overheating only in the outer areas, where the heat cannot be transported into the sheet in a sufficient manner. Figure 7c shows a picture of a heated sheet where the signs of overheating (unwetted, brittle, white fiber rovings) at the edges are very prominent (dashed region), and, also, the still consolidated regions at the clamps can clearly be located (dotted region). The center of the sheet exhibits typical signs of deconsolidation.

**Figure 8:**
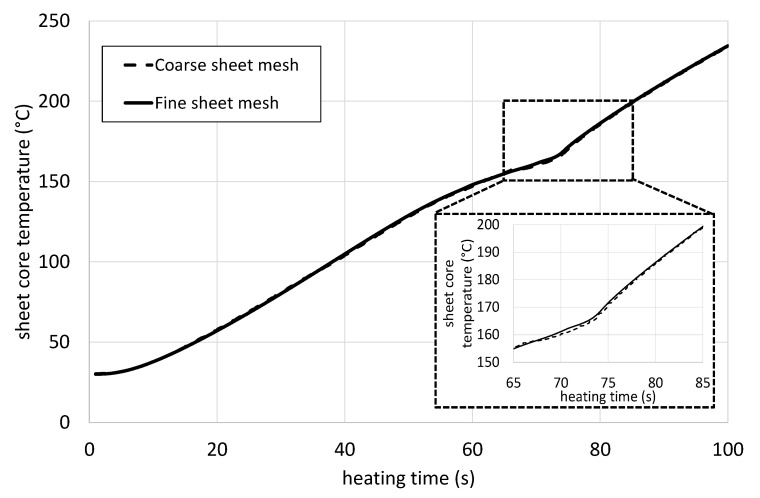
Comparison of two simulations of the heating of a sheet with a thickness of 4 mm at 150 mm sheet to heater distance and 80% heater power for two different meshes with a detailed view (dashed area) of the region of the melting point of the polypropylene matrix.

In order to validate the meshing result, the core temperature curves for the utilized fine and coarse mesh at the same heating conditions (4 mm sheet thickness, 80% heater power, and 150 mm sheet to heater distance) are plotted in Figure 8. The plots are congruent for the whole monitored heating time with one minor deviation at the melting point, which is highlighted in the figure as a zoomed plot. Since this discrepancy is insignificant, the simulations are commenced with the coarse mesh.

**Figure 9:**
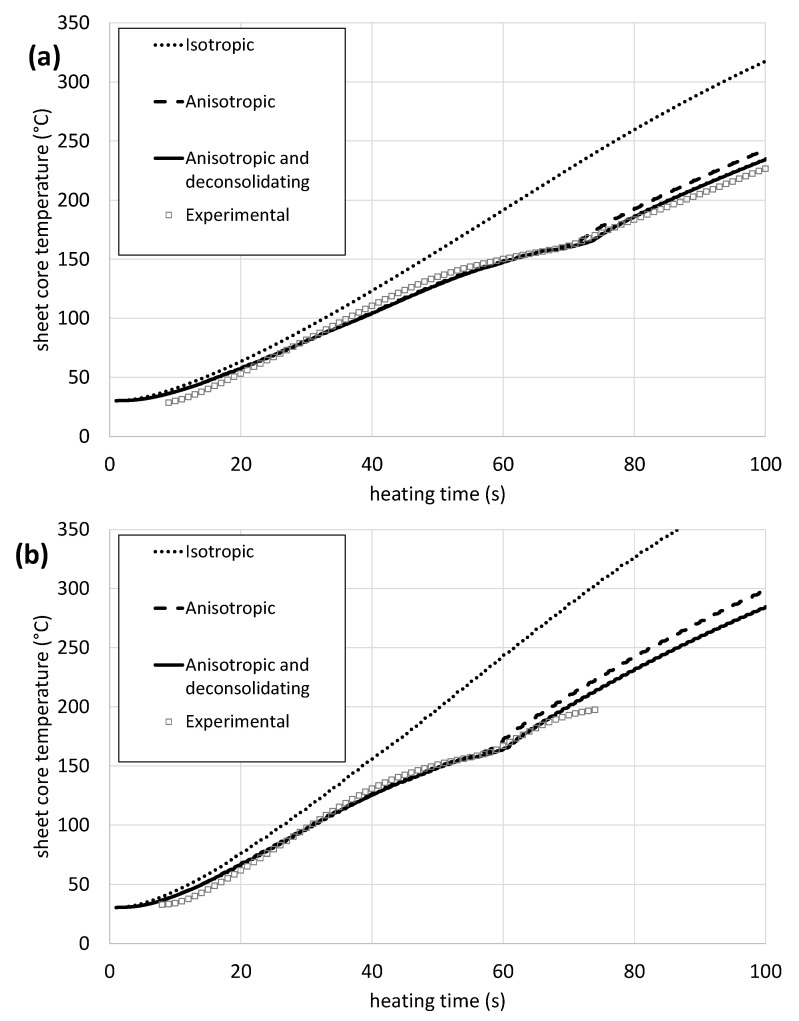
Result of heating tests of a 4 mm PP-GF sheet including the values derived from CFD-modeling. (**a**) Shows the values for 150 mm sheet to heater distance and 80% heater power and (**b**) shows the values for 150 mm sheet to heater distance and 100% heater power.

Figure 9 shows the measured core temperature of composite samples with 4 mm thickness and a sheet to heater distance of 150 mm on each side dependent on the heating time for different heater power settings and the corresponding model values derived from CFD-analysis. As expected, the sheet’s inner plies are heated faster with higher power. The *isotropic* material shows an almost linear heating behavior, that is clearly contradicted by the real course of the curve. The *anisotropic* case and the *anisotropic and deconsolidating* case are fairly close to each other, yet the major difference is the behavior after the transition temperature at 165 °C when thermal deconsolidation takes place. From there, the slope of the curve of the experiment is much closer to the model one, which considers thermal deconsolidation. The deviations at the end of the experiment are 7.6 °C (3.4% relative error), 15.9 °C (7% relative error), and 90.7 °C (40% relative error) at 80% power for the *anisotropic and deconsolidating*, *anisotropic*, and *isotropic* case, respectively. At 100% heater power, the surface of the composite is overheated quickly, which is why the experimental temperature in the core does not even reach temperatures above 200 °C before the test has to be aborted.

**Figure 10:**
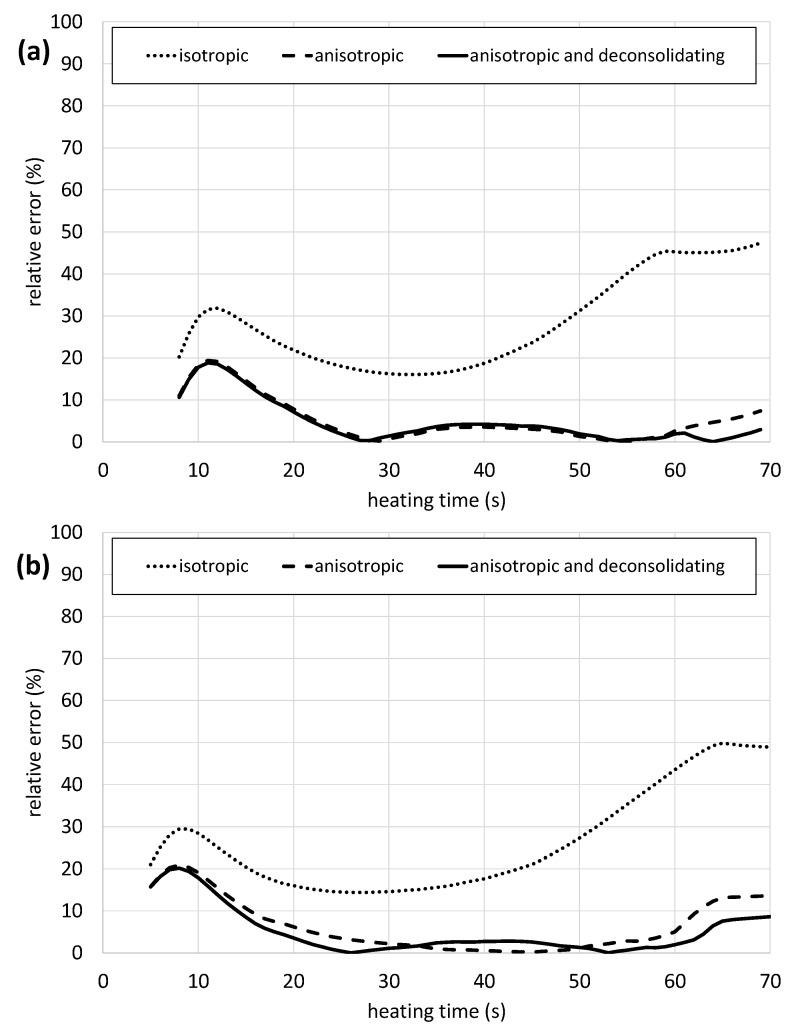
Relative errors of the CFD-model cases compared to the experimental values when heating a 4 mm PP/GF sheet at 100% heater power and 150 mm sheet to heater distance (**a**) and 100 % heater power and 200 mm sheet to heater distance (**b**).

Figure 10 shows the relative errors of the heating tests dependent on the heater distance setting for samples of 4 mm thickness, comparing them directly to the model values for all viewed material properties. Again, the most reliable results are derived from the *anisotropic and deconsolidating* case with lower deviations than the other material property cases for both heater distance settings. It is noticeable that at both heater distances, the error reaches a local maximum after a very short heating period. With time, the error drops to values of 0% for the *anisotropic* and the *anisotropic and deconsolidating* case when approaching the melting temperature. From there, the errors show a small incline, reaching up to 2.9% at 150 mm and 8.6% at 200 mm heater distance for the *anisotropic and deconsolidating* case, and 7.4% at 150 mm and 13.7% at 200 mm heater distance for the *anisotropic* case. The error in the *isotropic* case is close to 50% for both heater distance settings.

**Figure 11:**
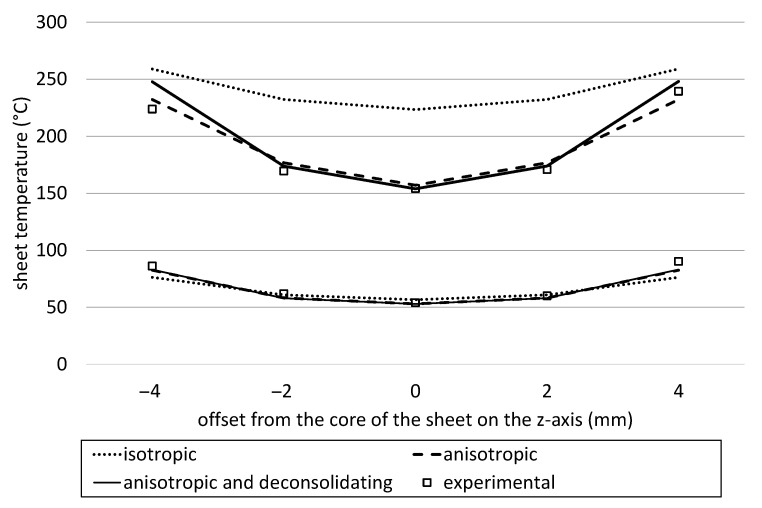
Temperature distribution through the thickness of a PP/GF sheet with a thickness of 8 mm that is heated with one active top and one active bottom heater running at 80% power at a distance of 100 mm at two heating times.

Figure 11 shows the temperature distribution at heating times of 20 s and 140 s from heating tests of the 8 mm material when only one single heater is activated on the top and the bottom heater bank with a power setting of 80% and a distance of 150 mm with the corresponding model values and the experimental data. This sheet was equipped with five thermocouples, that all passed the heating test unharmed. For this case, the whole cross-sectional temperature distribution can be investigated.

After 20 s of heating time, the temperature in the core of the sheet has not changed dramatically, and all of the models used deliver results within the accepted margin. With longer heating times, the *isotropic* case once again overshoots, especially in the center layers of the sheet. The *anisotropic* and the *anisotropic and deconsolidating* cases perform very well. The difference is not as clear, since the melting temperature has not been reached in the core after 140 s heating time. On the outside, the values are almost matching until the melting point is reached. In the experiments, this is also where thermal deconsolidation takes place first. The thermocouples tend to lose the perfect contact to the sheets, and the experimental values are not completely trustworthy any longer.

It is, also, notable that the measured temperature in the experiments is not symmetrical over the sheet thickness. The top surface shows higher readings than the bottom surface, even though the sheet to heater distance is the same. The reason for this behavior could be ascending heated air that is trapped under the upper heater bank which provides additional thermal input.

## 5. Discussion

The work shows the numerical and experimental heating analysis of thermoplastic composite sheets in a typical heating step of a thermoforming application. It can clearly be demonstrated by the experiments, that the temperature distribution through thickness and on the surface of the composite sheets is not uniform but rather strongly inhomogeneous. The only model that delivers satisfactory results, considers the anisotropic thermal conductivity, the anisotropic coefficient of linear thermal expansion, and the specific heat capacity at constant pressure to be temperature dependent.

Without those considerations, the models prove to be very inaccurate. Especially *isotropic* modeling leads to massive errors (40–50%) compared to the experiments. The consequence when using such models for process simulations would be significant under-heating of the parts. The sheets would be formed with a core temperature of up to 25 °C below the melting point, which will lead to inferior mechanical and optical properties.

When *anisotropy* is considered but deconsolidation is neglected, the simulation matches the experiments in a satisfactory way when the temperature of the sheet is below the transition temperature of the polymeric matrix. The knowledge of the temperature dependent specific heat capacity is a great asset as well, as it leads to a kink in the heating curve, that is also found in real experiments. From the point where the transition temperature is reached, a good estimate of the core temperature of the composite can only be made when using material models that consider thermal deconsolidation.

The combination of thermal deconsolidation and anisotropic thermal conductivity in CFD methods is new to the field and shows excellent agreement with actual experimental results.

In almost every numerical thermoforming study cited in the introduction, a uniform temperature distribution in the sheet is presumed before the forming step [19,20,22,23,24]. The knowledge of the real temperature distribution after heating could lead to significantly different results. Due to the inaccuracy of the heating modeling, not only the temperature distribution of the sheets is affected, in the long run this also influences cycle time calculations and over- and underheating issues and therefore the mechanical properties of the parts. In [28], an isotropic approach was used, which also matched the experimental data. This was enabled by numerical fitting of a parameter to calculate the heat transfer coefficient to equate the temperature predictions of the model and the results of the experiment.

**Figure 12:**
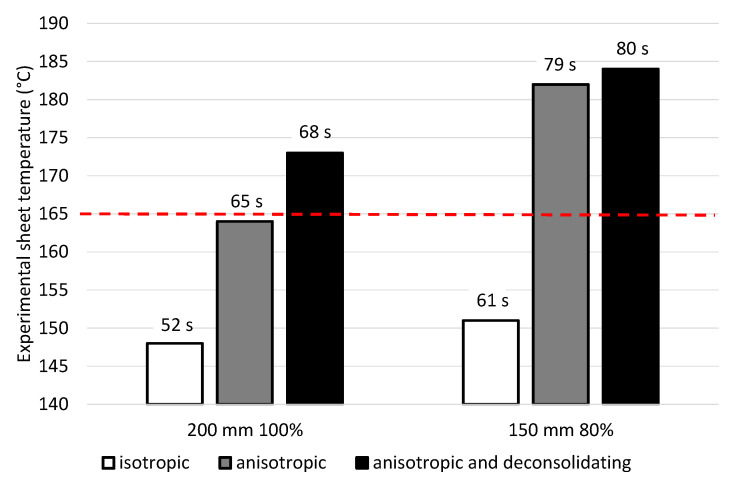
Experimental sheet core temperature at the time each model predicts 200 °C core temperature for two different settings (left: heater power of 100% at a distance of 200 mm; right: heater power of 80% at a distance of 150 mm) and a sheet of 4 mm thickness. The numbers above the bars are the experimental heating time at which the simulated data was retrieved. The red dashed line indicates the melting point of the PP matrix.

Judging from practical experience, PP/GF sheets are well formable at core temperatures of 200 °C. In industrial processes, core temperature tests are not available which is why, mostly, a trial-and-error principle is used. Figure 12 shows experimental sheet core temperatures at the time each model predicts 200 °C core temperature for 200 mm sheet to heater distance and 100% heater power and for 150 mm sheet to heater distance and 80% heater power. Furthermore, the figure shows the heating time that is considered for each case. The experimental heating time for those cases are 70 s and 87 s for a distance of 200 mm and 100% power and for 150 mm distance and 80% power, respectively. The red dashed line marks the transition temperature of the PP matrix. If these models were used in industrial processes, the sheets would not be formable for the *isotropic* case. The *anisotropic* material is a borderline case at the first setting and would be formable at the second one. Only the *anisotropic and deconsolidating* case is within the forming window of the PP matrix for both settings. The deviation in the first case looks large, yet the heating time is just 2 s shorter than the true experimental value to reach a temperature of 200 °C in the core.

This work yields the possibility to determine the temperature and volume of a composite sheet in every processing step. The future scope is to use the full potential of the model presented in this study to simulate the entire thermoforming process from the heating step to transportation and forming of the sheets to cooling and demolding of the final part.

## 6. Conclusions

This work shows how the use of novel, state-of-the-art material models for the temperature dependent anisotropic thermal conductivity and the anisotropic coefficient of linear thermal expansion can influence the results of CFD-modelling of thermoforming applications for thermoplastic composite materials in comparison to much simpler models. Since these materials must fulfil the highest standards in terms of mechanical behavior and aesthetics, precise process modeling is demanded. The numerical simulation is validated with experiments in which PP/GF sheets are heated in a laboratory thermoforming machine using different sheet to heater distances, heater power settings, and sheet thickness.

It can be shown that the anisotropic effects of thermal deconsolidation strongly influence the thermal conductivity and the linear coefficient of thermal expansion. This behavior can solely be simulated using the presented temperature dependent models, as the resulting comparisons between simulation and experimental data prove. The error in the heating time prediction can be lowered to under 3% for thick composite sheets. Using simpler models leads to unacceptable errors and inefficient processes. The heating time estimation herein delivers an error of over 25%, compared to experimental data. This work thus presents a powerful tool for thermoforming simulations and will help to improve processes and products.

## Figures and Tables

**Table 1:** Studied material behaviors and the corresponding material properties used for modeling.

Name	Linear Thermal Expansion	Specific Heat Capacity	Thermal Conductivity
isotropic	isothermal and isotropic	isothermal	isotropic and isothermal
anisotropic	isothermal and anisotropic	temperature dependent	anisotropic
anisotropic and deconsolidating	anisotropic and temperature dependent	temperature dependent	anisotropic and subject to thermal deconsolidation

**Table 2:** Material data used for the modeling of the anisotropic thermal conductivity and the deconsolidation volume [29,30].

Parameter	Description	Value
φ	Initial fiber volume fraction	0.47
k_v_	Thermal conductivity (voids)	0.026 W m^−1^ K^−1^
k_F_	Thermal conductivity (fibers)	1.03 W m^−1^ K^−1^
k_P_	Thermal conductivity (polymer)	0.20 W m^−1^ K^−1^
a	Unit cell edge length	4.1 mm
b	Roving width	4.0 mm
ρ_F_	Fiber density	2.5 g cm^−3^
α_F_	Coefficient of linear thermal expansion of the fibers	5 × 10^−6^ K^−1^
α_P_	Coefficient of linear thermal expansion of the polymer	1.6 × 10^−4^ K^−1^
m_A_	Area density of the textile	600 g m^−2^
v_m0_	Initial matrix volume fraction	0.53
m	Power law index, dependent on fiber configuration	10.2
p	Pressure at deconsolidation	101,000 Pa
p_0_	Initial pressure	202,000 Pa
T_0_	Initial temperature	298.15 K
$\varphi_{0}$	fiber volume fraction in the relaxed state	0.3
C_c_E_f_	Material constant multiplied by the fiber bending modulus	350 Pa

**Table 3:** Mesh statistics of the CFD model for the heaters and the clamping frame and two different element counts at the sheet (Avg. = Average value; σ = standard deviation).

	Mesh	Element Count	Skewness				Element Quality				Aspect Ratio			
			min	max	Avg.	σ	min	max	Avg.	σ	min	max	Avg.	σ
Heaters and Clamping Frame	fine	2.4 × 10^6^	7 × 10^−4^	0.84	0.26	0.11	0.2	0.99	0.81	0.09	1.2	8.8	1.91	0.42
Sheet	fine	84,000	10^−10^	10^−10^	10^−10^	0	1	1	1	10^−7^	1	1	1	0
	coarse	10,560	10^−10^	10^−10^	10^−10^	0	0.99	0.99	0.99	9 × 10^−8^	1.006	1.006	1.006	10^−7^

**Table 4:** Fit parameters to calculate the heater temperature for various heater settings.

Heater Setting (%)	Fit Parameter C	Fit Parameter D
20	28.07	83.51
30	35.04	111.41
40	41.73	138.19
50	47.50	161.30
60	51.59	177.66
70	57.13	199.85
80	60.63	213.83
90	64.57	229.63
100	68.17	244.05

## Data Availability

Not applicable.
